# Diabetes care knowledge and practice among primary care physicians in Southeast Nigeria: a cross-sectional study

**DOI:** 10.1186/s12875-020-01202-0

**Published:** 2020-07-01

**Authors:** Ejiofor Ugwu, Ekenechukwu Young, Marcellinus Nkpozi

**Affiliations:** 1 Enugu State University of Science and Technology, Enugu, Nigeria; 2 University of Nigeria Nsukka, Nsukka, Nigeria; 3 Abia State University Teaching Hospital, Aba, Nigeria

**Keywords:** Diabetes, Knowledge, Practice, Primary care, Physicians, Nigeria

## Abstract

**Background:**

Due to the perennial shortage of diabetes specialists, primary care physicians (PCPs) constitute the largest diabetes care manpower in Nigeria. However, the competence of the PCPs to undertake this task is not known. This study was aimed at evaluating diabetes care knowledge and practice among PCPs in Southeastern part of Nigeria.

**Methods:**

This was a cross-sectional study among PCPs in Southeastern Nigeria. Diabetes care knowledge and practices were evaluated using a structured self administered questionnaire.

**Results:**

A total of 64 PCPs with mean duration of practice of 17.3 ± 11.6 years completed the study. 65.6% were in private practice and 50% attended to between 11 and 20 persons living with diabetes (PLWD) weekly. Majority (78.1%) had not participated in any diabetes training since graduation from medical school and 79.9% were not aware of any diabetes clinical practice guideline.

The PCPs had adequate knowledge of classical symptoms of diabetes. However, they had very poor knowledge of glycemic thresholds for diagnosis of diabetes which was 26.6, 45.3 and 10.9% for fasting blood glucose (FBG), random blood glucose (RBG) and glycated hemoglobin (A1c) respectively.

We observed serious gaps in diabetes care practice such that only 18.8% of the respondents performed foot examination on newly diagnosed PLWD while 28.1 and 39.1% provided counseling on foot care and hypoglycemia respectively. Annual comprehensive foot examination was conducted by only 12.5%, none of the physicians ever screened for microalbuminuria and only 21.9% conducted annual dilated eye examination. Majority (57.8%) rated their confidence in prescribing insulin as “low” and only 23.4% had ever prescribed outpatient insulin for type 2 diabetes in their practice. Glycemic monitoring was largely limited to FBG and only 17.2% monitored A1c. Duration of practice more than 10 years (OR 10.1; P 0.034) and non participation in diabetes training (OR 6.5; P 0.027) were significant predictors of poor diabetes care knowledge.

**Conclusion:**

Diabetes care knowledge and practice were poor among PCPs in Southeast Nigeria. There is an urgent need to improve their capacity to provide diabetes care through periodic training.

## Background

Nigeria is one of the countries in sub-Saharan Africa (SSA) that are currently groaning under a rising prevalence of diabetes mellitus (DM). A recent meta-analysis reported that approximately 5.8% (about 6 million) of adult Nigerians are living with DM [1]. This figure has been likened to a tip of an iceberg as it is estimated that two-thirds of diabetes cases in Nigeria are yet undiagnosed [2]. This scenario which applies to most low and middle income countries of SSA has not only resulted to an increase in the burden of diabetes complications and deaths, but has also put a significant strain on the already weak health systems in this sub-region.

To arrest the current situation and improve the quality of diabetes care in SSA, concerted efforts to institute optimal diabetes care are required. These would entail the implementation of holistic strategies aimed at diabetes prevention through risk factor identification and lifestyle modification, as well as optimal glycemic control among subjects already living with diabetes. Landmark clinical trials have demonstrated that optimal glycemic control can prevent many of the diabetes related complications in both type 1 and type 2 diabetes [3, 4]. Implementing these strategies requires among other factors, a knowledgeable and motivated diabetes care workforce.

Diabetes specialists are arguably the most competent regarding diabetes care owing to their special training in this discipline. Regrettably this cadre of healthcare manpower is grossly in short supply in many countries of SSA. In Nigeria for instance, diabetes specialist to population ratio is estimated to be as low as 1 to 600,000. Furthermore, most of the specialists practice in tertiary healthcare centers which are usually located in the cities. Consequently, primary care physicians (PCPs) otherwise called general practitioners (GPs) constitute the largest diabetes care medical manpower in most SSA countries. Besides, a chronic care model built on primary healthcare systems in which a huge burden of care is placed on the PCPs has been advocated for low and middle income countries [5].

It has been demonstrated that the quality of diabetes care provided by PCPs is related to their diabetes knowledge [6]. Therefore, to be able to live up to this responsibility of providing optimal diabetes care, PCPs ought to possess sufficient diabetes care knowledge and skills. These include knowledge of diabetes risk factors, diagnosis, clinical and laboratory evaluation, metabolic monitoring, screening for diabetes related complications and prompt referral to specialists. There are limited data on the capacity of PCPs to provide optimal diabetes care in SSA. To our knowledge, no study has evaluated diabetes care knowledge and practice among PCPs in Nigeria. This study was therefore aimed at filling this gap.

## Methods

### Study design and subjects

This was a cross-sectional study conducted as pre-workshop evaluation during each of three diabetes training workshops for PCPs in Southeast Nigeria. The workshops which were sponsored by pharmaceutical companies and aimed at updating diabetes care knowledge among PCPs were part of advocacy efforts by the Diabetes Association of Nigeria (DAN) and took place between March and November 2018 at different locations including Fidelma Hotel Enugu, Ikenga Hotel Nsukka and Niger Foundation Hospital all in Enugu state, Southeast Nigeria. Letters of invitation were sent to PCPs in both public and private primary care centers across the 5 states of Southeastern Nigeria namely, Abia, Anambra, Ebonyi, Enugu and Imo States. Those who accepted to participate were provided free sponsorships to the workshops. The Research and Ethics Committee of Enugu State University Teaching Hospital approved the study while consent was obtained from each respondent prior to participation.

### The questionnaire

Diabetes knowledge and practices were evaluated using a self administered questionnaire (supplement 1) that was developed by the researchers for this purpose. It was structured to evaluate different domains of diabetes care including knowledge of diabetes risk factors, diagnostic criteria, pharmacotherapy of diabetes, counseling, glycemic monitoring, screening for diabetes complications and referral habits. The questions included both open-ended questions and multiple choice options. The Questionnaire was validated by two research experts; a consultant pediatrician and community health physician, both of whom are senior lecturers with Enugu State University of Science and Technology, Enugu. To test for the reliability of the questionnaire, a pilot study was conducted on 10 internship doctors at the Enugu State University Teaching Hospital by a split-half test method. Specifically, the items were split into two groups and then compared as if they were two separate administrations of the same survey. The questionnaire demonstrated good internal consistency of responses with Cronbach’s alpha coefficient of 0.753 and 0.835 for each spilt group, and a correlation between groups of 0.829, indicating a very strong reliability.

The respondents completed the questionnaires in the presence of a research assistant and submitted them immediately thereby eliminating the possibility of sourcing for answers elsewhere. Subjects who participated in more than one of the three workshops completed the questionnaire only once during their first workshop attendance.

### Assessment of knowledge of diabetes risk factors and diagnostic criteria

Participants were required to state at least three modifiable risk factors for type 2 diabetes and were judged knowledgeable if they mentioned obesity/overweight, physical inactivity and unhealthy diet. Knowledge of diabetes diagnosis was assessed by testing the participants’ knowledge of symptoms and glycemic thresholds based on the 1999 World Health Organization’s criteria [7]. The former was done by asking the respondents to state the classical symptoms of hyperglycemia. Those who stated at least three correctly including polyuria, polydipsia, polyphagia and weight loss were adjudged knowledgeable. Respondents were adjudged knowledgeable if they stated the diagnostic cut-offs as 7mmols/L for fasting blood glucose (FBG, 11.1mmols/L for random blood glucose (RBG) and 6.5% for glycated hemoglobin (A1c). Similarly, knowledge of prediabetes was evaluated by requesting the participants to state the glycemic range for impaired fasting glucose (5.6–6.9 mmol/L), impaired random glucose (7.8–11.0 mmol/L) and impaired A1c (5.7–6.4%). The respondents were also required to state any two diabetes clinical practice guideline (CPG) that they were aware of.

### Clinical and laboratory evaluation of persons living with diabetes

From a list of diagnostic tests including FBG, RBG, A1c and urine glucose, respondents were required to select the method(s) that they employed for diabetes diagnosis in routine clinical practice. The respondents were asked to select the clinical examinations that they routinely performed in newly diagnosed PLWD from a list that included blood pressure, weight, body mass index, waist circumference and foot examination. Furthermore, respondents were asked to indicate the laboratory work up that they conducted for evaluation of new DM patients from a list that included urinalysis, A1c, serum creatinine, lipid profile and electrocardiogram.

### Pharmacotherapy of diabetes and glycemic monitoring

From a list of the different classes of anti-diabetic medications, respondents were required to select the ones they commonly prescribed in the course of managing patients with diabetes. The list included biguanides, sulphonylureas (SUs), thiazolidinediones (TZDs), dipeptidyl peptidase 4 inhibitors (DPP-4i), alpha-glucosidase inhibitors (AGI) and insulin.

The respondents were further required to select the methods that they employed for glycemic and other monitoring of PLWD in routine clinical practice from a list that included FBG, PPG, A1c, urine glucose and weight.

### Counseling of persons living with diabetes

The participants’ practices regarding counseling of DM patients were evaluated by asking the respondents to indicate whether they routinely provided counseling to DM patients in different areas of disease management. The list included lifestyle modifications (diet and exercise), medication adherence, hypoglycemia, self blood glucose monitoring, and foot care education.

### Screening for diabetes complications

Participants answered “yes” or “no” to questions that evaluated their practices concerning annual screening tests for diabetes complications. These included annual dilated eye examinations, comprehensive foot examination, microalbuminuria testing, lipid profile measurements, serum creatinine and glomerular filtration rate (GFR) estimation.

### Challenges facing primary care physicians in diabetes care

We further asked the respondents to select what they considered significant challenges they encountered in caring for PLWD from a list of potential challenges drawn from the authors’ perceptions as well as from published literatures on similar subject [8–12]. Provisions were made for the respondents to state any other challenges not included in the options provided. Those responses were later examined and grouped into similar themes during analysis. Finally, the PCPs were asked to rate their confidence levels in three areas namely, selection of oral anti-diabetic medications, initiation of insulin, and managing diabetes generally.

### Data analysis

Data were extracted from the questionnaires into a personal computer and analyzed with the Statistical Package for Social Sciences (SPSS) (IBM version 23.0; SPSS Inc., Chicago, IL, USA). Only data from completely filled questionnaires were eligible for analysis. Categorical variables were presented as numbers and percentages while continuous variables were presented as means and standard deviations. Associations between participants’ characteristics and diabetes knowledge were tested using Chi-Square test while independent predictors of poor diabetes knowledge were determined by binary logistic regression. Statistical significance was established at *P* < 0.05.

## Results

Data from 64 out of 76 eligible participants (84.2%) were deemed suitable for analysis. Males were predominant (70.3%). The mean age of the participants was 45.5 ± 11.7 years. Majority of the respondents (65.6%) were in private practice and half of them attended to between 11 and 20 persons living with diabetes weekly. Majority (78.1%) had not participated in any diabetes training workshop since graduation from medical school. Table 1 shows the socio-demographic characteristics of the study participants.

**Table 1:** Demographic characteristics of the study population

Variable	Mean ± SD	Frequency	Percentage
**Age (years)**	45.5 ± 11.7		
≤ 40		28	43.8
> 40		36	56.3
**Gender**			
Males		45	70.3
Females		19	29.7
**Type of practice**			
Government		22	34.4
Private		42	65.6
**Location of practice**			
Urban		23	35.9
Rural		41	64.1
**Duration of Practice (years)**	17.3 ± 11.6		
< 10		24	37.5
≥ 10		40	62.5
**Number of DM patients seen/week**			
≤ 10		20	31.3
11–20		32	50.0
> 20		12	18.8
**Participation in DM training workshop since graduation from medical school**			
Yes		14	21.9
No		50	78.1

*DM* diabetes mellitus

### Knowledge of diabetes risk factors, symptoms and diagnostic criteria

**Fig. 1:**
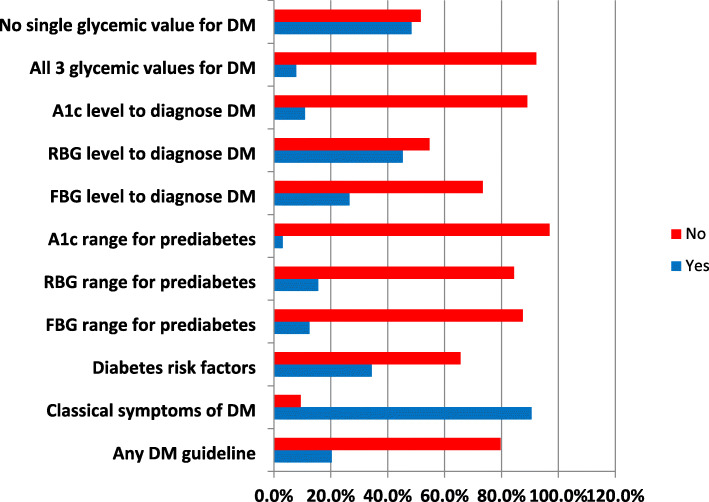
Knowledge of diabetes risk factors, symptoms and diagnosis among primary care physicians in Southeast Nigeria A1c = glycated hemoglobin, DM = diabetes mellitus, FBG = fasting blood glucose, RBG = random blood glucose

Adequate knowledge of classical symptoms of diabetes was possessed by 90.6% of the respondents. However, only 34.4% had sufficient knowledge of modifiable diabetes risk factors. Knowledge of glycemic cut-offs for diagnosis of diabetes was low at 26.6, 45.3 and 10.9% for FBG, RBG and A1c respectively. The PCPs generally lacked knowledge of prediabetes. Overall, only 5 out of 64 PCPs (7.8%) correctly knew all the three glycemic values for diagnosis of DM while 48.4% did not have correct knowledge of any one of the glycemic cut-offs for DM diagnosis. Fifty-one respondents (79.7%) were not aware of any diabetes management guideline (Fig. 1).

### Diabetes care practices among the study population

**Fig. 2:**
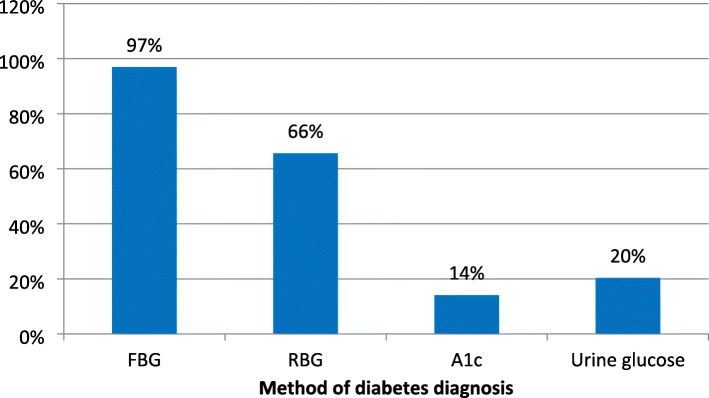
Methods of diagnosis of diabetes mellitus among primary care physicians in Southeast Nigeria. A1c = glycated hemoglobin, FBG = fasting blood glucose, RBG = random blood glucose

Figure 2 shows that FBG was the most frequently employed method of diabetes diagnosis (97%), followed by RBG (66%). Only about 14% of the PCPs reported that they had ever ordered A1c as part of diagnostic work up for diabetes while 20% diagnosed DM based on presence of glycosuria.

**Fig. 3:**
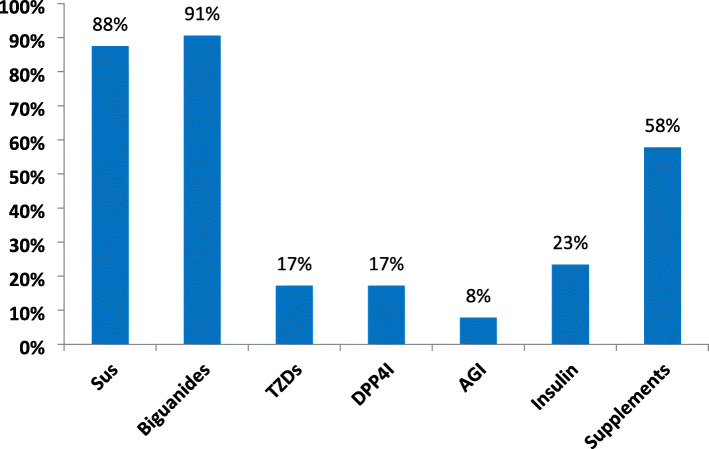
Prescription pattern of anti-diabetic medications among primary care physicians in Southeast Nigeria. SUs = sulphonylureas, TZDs = thiazolidinediones, DPP4I = dipeptidyl peptidase 4 inhibitors, AGI = alpha-glucosidase inhibitors

Anti-diabetic prescription pattern among the study participants is shown in Fig. 3. Sulphonylureas and biguanides were the most frequently prescribed medications. Only about 23% had ever prescribed insulin for outpatient with type 2 diabetes mellitus (T2DM) in their practice while 58% routinely prescribed multivitamin supplements alongside anti-diabetic medications.

Table 2 shows the practices of the study participants in various domains of diabetes care. For clinical assessment of newly diagnosed DM subjects, 100% of the respondents measured blood pressure. However, body mass index (BMI), waist circumference (WC) and foot examination were carried out by only 21.9, 14.1 and 18.8% respectively.

**Table 2:** Diabetes care practices among primary care physicians in Southeast Nigeria

Diabetes Care Domain	Number (***n*** = 64)	Percent (%)
**Clinical evaluation of new DM patients**		
Blood pressure	64	100.0
Body mass index	14	21.9
Waist circumference	9	14.1
Foot examination	12	18.8
**Laboratory evaluation of new DM patients**		
Urinalysis	49	76.6
Glycated hemoglobin (A1c)	17	26.5
Electrolytes, urea and creatinine	48	75.0
Lipid profile	44	68.8
Electrocardiogram	13	20.3
**Counseling provided to DM patients**		
Lifestyle modification	54	84.4
Hypoglycemia	25	39.1
Medication adherence	51	79.7
Self monitoring of blood glucose	19	29.7
Foot care education	18	28.1
**Glycemic and cardiometabolic monitoring**		
Fasting glucose	64	100
Post prandial glucose	19	29.7
Glycated hemoglobin (A1c)	11	17.2
Weight	20	31.3
**Screening for complications**		
Annual Serum creatinine	15	23.4
Annual lipid profile	18	28.1
Annual dilated eye examination	14	21.9
Annual microalbuminuria testing	0	0
Annual comprehensive foot examination	8	12.5
**Referral to diabetologist**		
For poor glycemic control	20	31.3
When complications are detected	29	45.3
When complications are advanced	50	78.1
**Others**		
Screen asymptomatic adults for diabetes in routine practice	37	57.8
Desire periodic diabetes management workshop	61	95.3

For laboratory evaluation of new DM subjects, majority of the respondents performed urinalysis, serum electrolytes and creatinine, and lipid profile. Electrocardiogram (ECG) and A1c were however hardly conducted as shown by only 20.3 and 26.5% of the PCPs respectively.

Majority of the respondents reported that they counseled patients on lifestyle modifications (84.4%) and medication adherence (79.7%). However, only 39.1 and 28.1% the study participants educated their patients on hypoglycemia and foot care respectively. All the respondents reportedly monitored FBG but PPG and A1c assessments were performed by only 29.7 and 17.2% respectively.

Screening for diabetes complications in asymptomatic subjects was an uncommon practice among the participants. Annual comprehensive foot examination was performed by only 12.5% of the respondents, and none of the PCPs ever requested microalbuminuria testing. While most of the PCPs (78.1%) referred their patients to diabetes specialists when they develop advanced complications, only 31.3% would do so for poor glucose control. Nearly all the respondents (95.3%) desired to receive periodic diabetes management training.

**Fig. 4:**
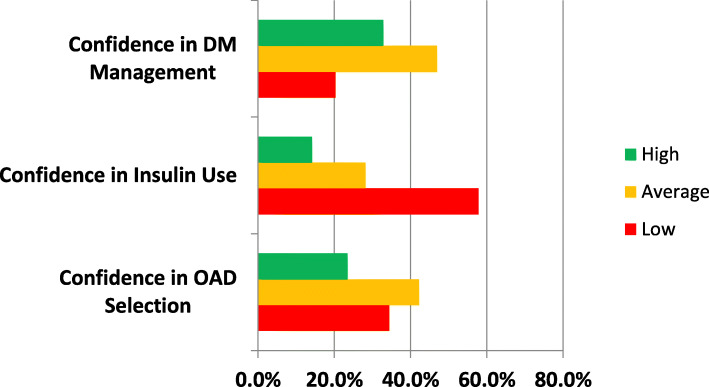
Self confidence rating on diabetes care by primary care physicians in Southeast Nigeria. DM = diabetes mellitus, OAD = oral anti-diabetic drugs

Figure 4 shows the self confidence rating of the PCPs in diabetes management. 34.4% of the PCPs rated themselves low in ability to correctly select oral anti-diabetic medications. On insulin initiation, majority (57.8%) rated their confidence level low. Confidence in managing diabetes generally was rated as average by 46.9% of the respondents.

### Challenges confronting primary care physicians in diabetes care

**Fig. 5:**
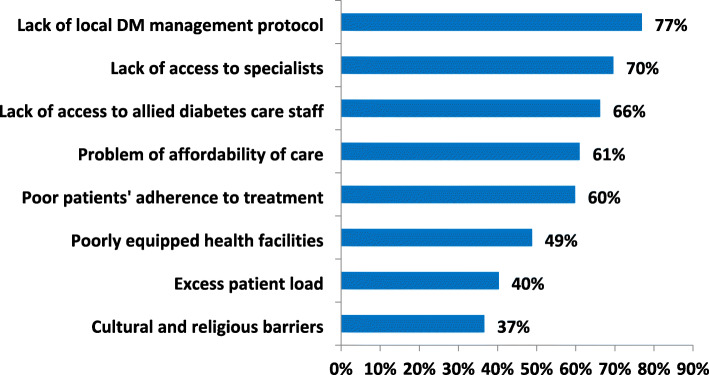
Challenges of diabetes care faced by primary care physicians in Southeast Nigeria. DM = diabetes mellitus

The PCPs identified absence of local diabetes clinical practice guidelines (CPG) as the greatest challenge they face in managing people with diabetes. Lack of access to diabetes specialists and allied healthcare professionals were identified by 70 and 66% of the respondents respectively (Fig. 5).

### Predictors of poor diabetes knowledge

Duration of medical practice more than 10 years (OR 10.1; P 0.034) and non participation in diabetes training (OR 6.5; P 0.027) were significant predictors of poor diabetes knowledge in the study population (Table 3).

**Table 3:** Predictors of poor diabetes knowledge among primary care physicians in Southeast Nigeria

	Knowledge of DM Diagnosis				
	Poor	Good	*P* value	OR	95% C. I for OR
***Age***					
> 40 years	33 (66.0)	3 (21.4)	0.35	2.49	0.37–16.92
≤ 40 years	17 (34.0)	11 (78/6)			
***Gender***					
Males	36 (72.0)	9 (64.3)	0.63	1.51	0.28–8.06
Females	14 (28.0)	5 (35.7)			
***Duration of Practice***					
> 10 years	38 (76.0)	2 (14.3)	**0.03**	10.11	1.20–85.41
≤ 10 years	12 (24.0)	12 (85.7)			
***Type of Practice***					
Government	19 (38.0)	3 (21.4)	0.70	1.47	0.21–10.06
Private	31 (62.0)	11 (78.6)			
***Location of Practice***					
Urban	33 (66.0)	11 (78.6)	0.35	2.62	0.34–20.01
Rural	17 (34.0)	3 (21.4)			
***Workshop participation***					
Never	44 (88.0)	6 (42.9)	**0.03**	6.55	1.24–34.67
Yes	6 (12.0)	8 (57.1)			

*DM* diabetes mellitus

## Discussion

In spite of the strategic importance of primary care physicians as major providers of diabetes care in Nigeria, little is known about their competence to perform this crucial role. This study was conducted to evaluate diabetes care knowledge and practice among PCPs in Southeast Nigeria.

### Knowledge of diagnostic criteria for diabetes and Prediabetes

Our study uncovered a low level of diabetes care knowledge among the PCPs. Although FBG was the most frequently employed method of diabetes diagnosis by the study participants, yet, nearly three-quarters of the PCPs lacked correct knowledge of diabetic range FBG. It was observed that only 5 out of the 64 PCPs (7.8%) correctly knew all the three glycemic cut-offs and nearly half did not have correct knowledge of any single glycemic value for diagnosis of DM. This finding is worrisome as it suggests the existence of a high rate of diabetes misdiagnosis in Southeast Nigeria. There are no known studies on this subject in Nigeria to draw comparisons from. Nonetheless, we hypothesize that this finding is likely to apply nationwide rather than a regional phenomenon peculiar to the Southeastern part of the country. If our speculation is true, the public health impact of this observation is expected to be grave in Nigeria since the bulk of DM diagnosis and management rests on the shoulders of PCPs.

Our findings are similar to those of other researchers in Cameroon [8], China [9] and Sri Lanka [10] who also reported significant deficits in diabetes knowledge among PCPs. In contrast, 66.6 and 71.7% of primary care doctors in Egypt and Saudi Arabia respectively knew the correct diagnostic criteria for DM [11, 12]. The low level of diabetes knowledge in this study could be attributed partly to lack of continual medical education (CME). In support of this notion, we observed that respondents who had participated in diabetes update course were significantly more knowledgeable than those who had not. Continual medical education has been demonstrated to significantly improve both diabetes knowledge and practice among PCPs [11–13]. It is noteworthy that nearly four-fifth of our respondents had never participated in any diabetes training since graduation from medical school. We also observed that doctors with shorter duration of practice were more knowledgeable than the longer practicing ones, similar to the observation in Saudi Arabia [12]. This could be due to the fact that more recent medical graduates are probably more abreast with current trends in diabetes care than older ones. In contrast, diabetes knowledge and practice confidence were unrelated to the number of practice years in a study among rural doctors in Australia [14].

Similar to a recent observation in a neighboring country [8], knowledge of pre-diabetes was almost non-existent among our respondents. This means that majority of the PCPs would be unable to recognize this important stage in the evolution of diabetes which has been demonstrated to be associated with macrovascular diseases including stroke, coronary artery disease and peripheral artery disease [15]. Cardiovascular diseases (CVDs) are the commonest causes of death globally. Low and middle income countries of SSA are ill-equipped to tackle the rising menace of CVDs thereby making their prevention a compelling option. It is therefore imperative that PCPs are capacitated to recognize CVD risk factors such as prediabetes. Importantly, appropriate lifestyle interventions in prediabetic subjects have been demonstrated to be capable of reversing or delaying the onset of diabetes [16]. Regrettably too, only about a third of our respondents had good knowledge of modifiable diabetes risk factors including unhealthy diet, physical inactivity and overweight/obesity. It is therefore deducible that most of the PCPs lacked sufficient knowledge to contribute significantly to diabetes prevention in Southeast Nigeria. This situation is unfortunate for a country with a relentless increase in the prevalence of diabetes.

### Diabetes care practices among the primary care physicians

Sulphonylureas (SUs) and biguanides were the most frequently prescribed anti-diabetic medications among our study participants. Only few PCPs reported ever prescribing other classes of anti diabetic medications to their patients. This finding which is similar to those reported from Cameroon [8] and Sri Lanka [10] may be attributable to inadequate knowledge of diabetes pharmacotherapy. In one study, general physicians identified pharmacological management of diabetes as their greatest learning needs [14]. Other plausible reasons for our observation include lack of availability and higher cost of the newer anti diabetic medicines. On the other hand, over half of our respondents routinely prescribed multivitamin supplements to their patients. Professional guidelines do not support routine multivitamin supplementation in PLWD due to lack of evidence to justify such practice [17]. Instead, this practice is largely driven by profit oriented pharmaceutical industries and constitute needless financial burden on the patients, most of whom are already resource constrained. It is therefore high time unhelpful practices like this are discouraged so that healthcare resources could be better utilized.

Less than a quarter of the respondents ever prescribed insulin for out-patient care of T2DM subjects and majority rated their confidence to initiate insulin therapy as “low”. This inertia to prescribe insulin was observed among PCPs in many other studies [8, 13, 14]. Insulin remains the most effective anti-diabetic medicine that may be required in the time course of T2DM management to achieve good glycemic control. With this high degree of inertia to employ insulin by the PCPs, patients who are not optimally controlled on oral medications would therefore remain in chronic hyperglycemia which is an established risk factor for many of the diabetes complications.

Notable inadequacies in the evaluation of newly diagnosed DM patients and screening for diabetic complications were observed among our respondents. Although blood pressure measurements were reportedly performed by all the PCPs, assessment of markers of adiposity (waist circumference, BMI) and foot examination were hardly carried out. Systemic manifestations of diabetes complications often present first in the foot [18]. It is therefore very likely that early foot lesions in PLWD would go unrecognized by the PCPs until ulceration develops. Unfortunately too, less than a third of the PCPs provided foot care education to their patients and annual comprehensive foot examination was conducted by only a few physicians. Could this undesirable practice largely account for the high burden of diabetic foot ulcerations (DFU) and lower extremity amputations in Nigeria? [19, 20]. In a recent multicenter study in Nigeria in which 85% of the subjects hospitalized for DFU had never received foot care education prior to development of ulcers, 90% of the patients were primarily managed by PCPs [20]. Screening for other DM complications including retinopathy and nephropathy were also uncommon practices among the PCPs. In fact, no single physician ever requested microalbuminuria testing. It’s therefore logical to infer that patients managed by these PCPs are at high risk of progression of DM complications with resultant disability and/or death. In contrast to our observations, over 80% of PCPs routinely conducted periodic screening for diabetes complications in Sri Lanka and South Africa [10, 21]. Inadequate knowledge of diabetes clinical practice guidelines (CPG) and lack of equipments may explain the observed low quality of diabetes care practices among our respondents. A positive correlation between knowledge of diabetes CPG and better diabetes care practices among GPs has been demonstrated [12, 21]. Only 20.3% of our respondents were aware of any diabetes CPG, compared to Australia and South Africa where knowledge of diabetes CPG were reported in 95.6 and 92% of general physicians respectively [14, 21]. It is noteworthy that absence of local diabetes management protocol was identified by our respondents as the commonest challenge they faced in the care of PLWD in their practice.

Majority of the respondents counseled their subjects on lifestyle modifications and medication adherence. This is commendable since appropriate lifestyle changes are crucial part of proper diabetes care which have been shown to improve glycemic control [22]. However, most of them provided no education regarding hypoglycemia. This is in spite of the fact that sulphonylureas which have a high propensity to cause hypoglycemia were among the most prescribed medications in this study. Hypoglycemia is a life threatening complication of anti-diabetic medications that should be avoided at all cost. It is associated with cognitive impairment, permanent brain damage, cardiac arrhythmias and death [23].

Glycemic monitoring was largely limited to fasting glucose while post prandial glucose and A1c were rarely assessed. Although fasting glucose control is important, post prandial glycemia has been shown to also impact significantly on A1c and is associated with chronic diabetes complications [24, 25]. The limitation of both FBG and PPG is that they only assess short term glycemic excursions. Glycated hemoglobin therefore remains the gold standard for assessment of long term glycemia in subjects with diabetes and international guidelines recommend its assessment at least two to four times annually [17]. High A1c values have been shown to correlate significantly with the development of nearly all chronic complications of diabetes [26]. Regrettably, less than one-fifth of our respondents assessed A1c in their practice. The reasons for this pitfall are likely multifactoral and may include lack of facility and attendant high cost of A1c testing compared to blood glucose. Indeed, poor knowledge of the healthcare professionals on the utility of A1c in diabetes care may also be contributory.

One of the observations made in this study was the high threshold to refer patients to diabetes specialists. Less than a third of our respondents would refer their patients to specialists for poor glucose control and majority would rather wait until complications are detected or are advanced. This is in contrast to the practice in Saudi Arabia where 64% of GPs preferred to refer all newly diagnosed PLWD to specialists to initiate treatment [12]. The reason for this reluctance to refer patients to specialists is unclear. However since majority of our respondents were in private practice which are usually profit-driven, it may not be unconnected with the fear of losing clientele base and possible financial loss. Other plausible explanations include lack of ready access to specialists and concerns about affordability of specialist care, both of which were identified by majority of the PCPs as significant challenges to diabetes care in their practice. Whatever the reasons may be, this type of practice invariably does more harm than good to the patients and should be strongly discouraged.

### Strengths and limitations

To our knowledge, this is the first study to assess diabetes care knowledge and practice among general practitioners in Nigeria. However, the number of PCPs who participated in this study is insufficient to permit generalization of our findings to the rest of the country. Secondly, like all studies of this nature, we relied on the integrity of the respondents to evaluate their practice. Therefore, it is not unlikely that their reported practices might differ from actual practice.

## Conclusion

This study has exposed serious gaps in knowledge and practices in diabetes care among primary care physicians in Southeast Nigeria. Majority of the practitioners lacked adequate knowledge of correct diagnostic criteria for pre-diabetes and diabetes. Choices of anti-diabetic medications were largely limited to sulphonylureas and metformin and glycemic assessments were grossly inadequate. The physicians provided counseling on lifestyle modifications and medication adherence but not on hypoglycemia and foot care. Screening for diabetic complications was hardly performed and regrettably, the PCPs had high threshold to refer their patients for specialist care despite poor glucose control. Majority of the PCPs had never had further training on diabetes care since they graduated from medical school and were ignorant of any standards of care for diabetes. Absence of local diabetes management protocol, lack of access to specialists and allied diabetes care professionals, affordability of care and poorly equipped care centers were the commonest challenges to diabetes care reported by the PCPs.

Since PCPs constitute the largest diabetes care workforce in Nigeria, effort to address these observed gaps in diabetes knowledge, attitude and practice are a matter of urgent importance. In view of this, we make the following recommendations:

1. Periodic training of primary care physicians on diabetes care. This could be built into the mandatory annual CME required for renewal of practicing license.
2. Development of a simplified diabetes management algorithm by the Diabetes Association of Nigeria in collaboration with ministries of health which should be made available to all primary care doctors.
3. Establishment of mentorship programs whereby a group of PCPs in a defined local community are assigned to a diabetes specialist for prompt access to professional advice.

## Supplementary information

**Additional file 1: Supplement 1.** Questionnaire to evaluate knowledge and practice of diabetes care among general practitioners in Southeast Nigeria. This supplementary file contains the questionnaire that was used to evaluate knowledge and practice of diabetes care among general practitioners in Southeast Nigeria.

## Data Availability

The datasets used and/or analyzed during the current study are available from the corresponding author on reasonable request.

## Abbreviations

A1c: Glycated hemoglobin
AGI: Alpha-glucosidase inhibitors
BMI: Body mass index
DAN: Diabetes Association of Nigeria
DM: Diabetes mellitus
DPP4-I: Dipeptidyl peptidase 4 inhibitors
ECG: Electrocardiogram
FBG: Fasting blood glucose
PCP: Primary care physicians
PLWD: Person living with diabetes
RBG: Rrandom blood glucose
SPSS: Statistical package for social sciences
SSA: Sub-Saharan Africa
SU: Sulphonylureas
T2DM: Type 2 diabetes mellitus
TZD: Thiazolidinediones
WC: Waist circumference
