# Supplementary material for: Diabetes care knowledge and practice among primary care physicians in Southeast Nigeria: a cross-sectional study
Source: BMC Fam Pract. 2020 Jul 1;21:128. doi: 10.1186/s12875-020-01202-0 (PMC7330977; doi:10.1186/s12875-020-01202-0)
Supplement: Supplementary file 1 — Additional file 1: Supplement 1. Questionnaire to evaluate knowledge and practice of diabetes care among general practitioners in Southeast Nigeria. This supplementary file contains the questionnaire that was used to evaluate knowledge and practice of diabetes care among general practitioners in Southeast Nigeria. [file 12875_2020_1202_MOESM1_ESM.docx]

**QUESTIONNAIRE TO EVALUATE KNOWLEDGE AND PRACTICE OF DIABETES CARE AMONG GENERAL PRACTITIONERS IN SOUTHEAST NIGERIA**

**(A): SOCIO-DEMOGRAPHIC INFORMATION**

**1. Age** (years) ………………..

**2. Gender**. Male [ ] Female [ ]

**3. Duration of Practice as a Doctor** (years) ……………………….

**4. Type of Practice Facility**. Government Hospital [ ] Private Hospital [ ]

**5. Location of Practice** (a) Urban [ ] (b) Rural [ ]

**6. In your practice, how many diabetes patients do you attend to weekly?**

(a) ≤ 10 [ ] (b) 11 – 20 [ ] (c) 21-30 [ ] (d) 31-40 [ ] (e) > 40 [ ]

**7. Do you have facility for glycated hemoglobin (HbA1c) in your practice?**

(a)Yes [ ] (b) No [ ]

**8. Since graduation, when last did you participate in diabetes education workshop?**

(a) Never [ ] (b) Past 1-2 years [ ] (c) Past 3-4 yrs [ ] (d) ≥ 5 yrs [ ]

**(B): KNOWLEDGE ABOUT DIABETES**

**9. List the 3 classical symptoms of diabetes? a. …………… b. ………… c. …………**

**10. List 3 modifiable risk factors for diabetes a. …………… b. ……………c. …………..**

**11. State the glycemic cut-off values for diagnosis of prediabetes and diabetes in the table below**

| **Parameter** | **Range for Pre-diabetes** | **Range for Diabetes** |
| --- | --- | --- |
| **Fasting blood glucose (mg/dl)** |  |  |
| **Random blood glucose (mg/dl)** |  |  |
| **Glycated Hemoglobin HbA1c (%)** |  |  |

**12. Do you know of any professional guideline for managing DM?**  (a) Yes [ ] (b) No [ ]

**13. If yes, state two guidelines that you know:**

**(a). ………………………………………………………………………………**

**(b). …………………………………………………………………………..….**

**(C): DIABETES CARE PRACTICE**

**14. In your practice, which of these tests do you employ in making a diagnosis of DM (tick ALL applicable options) ?**

(a) FBS [ ] (b) RBS [ ] (c) Urine glucose [ ] (d) OGTT [ ] (e) HbA1c [ ]

**15. Which of these CLINICAL examinations do you routinely perform as part of evaluation of newly diagnosed type 2 DM in your practice?**

1. **Blood Pressure** (a) Yes [ ] (b) No [ ]
2. **Body mass index** (a) Yes [ ] (b) No [ ]
3. **Waist circumference** (a) Yes [ ] (b) No [ ]
4. **Foot examination** (a) Yes [ ] (b) No [ ]
5. **Dilated eye examination** (a) Yes [ ] (b) No [ ]

**16. Which of these LABORATORY tests do you routinely order as part of evaluation of newly diagnosed type 2 DM in your practice?**

1. **Urine Analysis** (a) Yes [ ] (b) No [ ]
2. **HbA1c** (a) Yes [ ] (b) No [ ]
3. **Lipid profile** (a) Yes [ ] (b) No [ ]
4. **Electrolyte urea and creatinine** (a) Yes [ ] (b) No [ ]
5. **Electrocardiogram** (a) Yes [ ] (b) No [ ]

**17. Indicate which of these areas of diabetes care that you routinely provide counseling to diabetic patients in your practice**

1. **Lifestyle modification** (a) Yes [ ] (b) No [ ]
2. **Medication adherence** (a) Yes [ ] (b) No [ ]
3. **Hypoglycemia** (a) Yes [ ] (b) No [ ]
4. **Lipid profile** (a) Yes [ ] (b) No [ ]
5. **Self blood glucose monitoring** (a) Yes [ ] (b) No [ ]
6. **Foot care** (a) Yes [ ] (b) No [ ]

**18. Select ALL classes of anti-diabetic medications that you have ever prescribed for out-patients with type 2 diabetes in your practice.**

1. **Sulphonylureas** (a) Yes [ ] (b) No [ ]
2. **Biguanides** (a) Yes [ ] (b) No [ ]
3. **Thiazolidinediones** (a) Yes [ ] (b) No [ ]
4. **Alpha glucosidase inhibitors** (a) Yes [ ] (b) No [ ]
5. **DPP4 inhibitors** (a) Yes [ ] (b) No [ ]
6. **Insulin** (a) Yes [ ] (b) No [ ]
7. **Multivitamin supplemets** (a) Yes [ ] (b) No [ ]

**19. Which of these glycemic and metabolic parameters do you regularly monitor in diabetic patients in your practice (tick ALL that are applicable)**

1. **Fasting blood glucose** (a) Yes [ ] (b) No [ ]
2. **Post meal blood glucose** (a) Yes [ ] (b) No [ ]
3. **HbA1c** (a) Yes [ ] (b) No [ ]
4. **Weight** (a) Yes [ ] (b) No [ ]

**20. Which of the following tests do you routinely conduct to screen for diabetes complications in your practice?**

1. **Annual dilated eye examination** (a) Yes [ ] (b) No [ ]
2. **Annual urine micro albumin** (a) Yes [ ] (b) No [ ]
3. **Annual comprehensive foot examination** (a) Yes [ ] (b) No [ ]
4. **Annual Lipid profile** (a) Yes [ ] (b) No [ ]
5. **Annual serum electrolyte urea and creatinine** (a) Yes [ ] (b) No [ ]

**21. Which of the following constitute sufficient indication for referral of diabetic patients to specialist endocrinologists in your routine practice (tick ALL that are applicable)?**

1. **Poor glucose control** (a) Yes [ ] (b) No [ ]
2. **As soon as chronic complications are noticed** (a) Yes [ ] (b) No [ ]
3. **When complications are advanced** (a) Yes [ ] (b) No [ ]
4. **I do not refer patients to specialist for any reason**  (a) Yes [ ] (b) No [ ]

**(D): BARRIERS TO DIABETES CARE IN PRACTICE**

**22. Which of these constitute significant challenges to the care of diabetic patients in your practice? (tick all that are applicable)**

a. Affordability of care

b. Lack of equipments

c. Lack of access to diabetes specialists

d. Lack of local diabetes management protocol

e. Lack of access to allied diabetes care professionals (educators, nutritionists, podiatrists, etc

f. Cultural and religious interferences

g. Excess workload

h. Poor patients’ adherence to treatment

h. Others (specify) ………………………………………………….

**(E). OTHERS**

**23. In your practice, do you routinely screen asymptomatic adults for diabetes mellitus?**

(a) Yes [ ] (b) No [ ]

**24. On a scale of 1-3, rate your knowledge in selecting appropriate oral medications for patients with Type 2 DM ………..**

**25. On a scale of 1-3, rate your confidence in initiating insulin for out-patients with type 2 DM ………..**

**26. On a scale of 1-3, rate your confidence in managing diabetes generally ………….**

**27. Are you willing to receive periodic diabetes training?** (a) Yes [ ] (b) No [ ]

**THANK YOU.**
